# Public Perception of Clinical Trials and Its Predictors Among Polish Adults

**DOI:** 10.3390/jcm14103279

**Published:** 2025-05-08

**Authors:** Alicja Kozakiewicz, Joanna Mazur, Monika Szkultecka-Dębek, Maciej Białorudzki, Zbigniew Izdebski

**Affiliations:** 1 Department of Humanization of Medicine and Sexology, Collegium Medicum, University of Zielona Góra, 65-046 Zielona Góra, Poland; j.mazur@inz.uz.zgora.pl; 2 Faculty of Applied Sciences, University of Social Sciences, 00-820 Warsaw, Poland; mszkultecka-debek@san.edu.pl; 3 Faculty of Education, University of Warsaw, 00-927 Warsaw, Poland; m.bialorudzki@uw.edu.pl; 4 Department of Biomedical Aspects of Development and Sexology, Faculty of Education, University of Warsaw, 00-927 Warsaw, Poland; zbigniew.izdebski@uw.edu.pl

**Keywords:** clinical trials, clinical research, patient motivation, patient recruitment, public engagement, socioeconomic factors, Poland

## Abstract

**Background/Objectives**: Public perception of clinical trials (CT) in Poland remains underexplored. This study aims to assess attitudes towards clinical trials, to identify key sociodemographic and health-related predictors of participation willingness, and to evaluate the perceived health impact (CT-PHI) associated with participants’ involvement in trials. **Methods**: A cross-sectional online survey was conducted (2–20 March 2022) among 2050 Polish adults who had benefited from medical care in the past 24 months. This study examined sociodemographic factors, health-related factors (self-perceived health, EQ-5D-5L, the level of awareness of patient rights, use of public vs. private healthcare, adherence), and motivations for participation in trials (health-related, financial, and altruistic). Multivariate logistic and generalized linear models identified predictors of participation willingness and CT-PHI variability. **Results**: Overall, 56.3% of the respondents expressed a willingness to participate in clinical trials. The main motivation was health improvement (45.8%), followed by financial incentives (23.2%) and altruism (22.7%). Those driven by health reasons showed the highest sensitivity to demographic and health-related factors. In this group, higher CT-PHI scores were associated with older age (66+: B = 1.24, *p* < 0.001), female gender (B = 0.41, *p* = 0.034), rural residence (B = 0.41, *p* = 0.033), dual use of public/private healthcare (B = 0.64, *p* = 0.001), adherence (B = 2.50, *p* < 0.001), and greater pain severity (moderate: B = 1.36, *p* < 0.001; severe: B = 0.91, *p* = 0.023). Socioeconomic factors played a greater role for financially motivated individuals, while altruistic participants showed the least variability in influencing factors. **Conclusions**: Willingness to participate in clinical trials in Poland is influenced by the motivation type. A patient-centered approach to recruitment strategies, considering diverse motivations and sociodemographic factors, is essential for optimizing participation in clinical trials.

## 1. Introduction

According to the definition in [1], clinical trials (CT) are carefully designed and verified analyses of new diagnostic and treatment methods that follow a pre-approved protocol. Furthermore, the Medical Research Agency, established in Poland by law in 2019 and responsible for the development of research in the field of medical sciences and health sciences, emphasizes that clinical trials are aimed at discovering or confirming the clinical and pharmacological (including pharmacodynamic) effects of one or more investigational medicinal products. CT are also performed with the aim of identifying adverse reactions and tracking the absorption, distribution, metabolism, and excretion of one or more investigational medicinal products, considering their safety and efficacy [2].

Poland represents a particularly compelling case study in the context of the public perception of clinical trials due to several inter-related historical and systemic factors. First, post-communist societies, including Poland, have historically exhibited a degree of institutional distrust, especially toward scientific and medical authorities [3]. This lingering skepticism can influence public attitudes toward participation in clinical research, particularly when it comes to issues of informed consent, safety, and transparency. Second, the Polish healthcare system, although publicly funded and universally available, often struggles with underfunding, limited access to specialist care, and long waiting lists [4], which may impact both the perceived risks and potential benefits of participating in clinical trials.

Despite these challenges, Poland, along with the rest of Central and Eastern Europe (CEE), has grown into a market leader in innovative bio-pharmaceutical commercial clinical trials (iBPCT). In 2019, it ranked 11th in the world in terms of iBPCT market share, and in 2014–2019, it recorded one of the largest increases in iBPCT market share in the world, ranking 5th behind China, Spain, South Korea, and Taiwan [5]. However, in spite of Poland’s growing role in the global market of innovative bio-pharmaceutical commercial clinical trials, a Polish study shows that public awareness of clinical trials remains relatively low. Although 86.5% of respondents had heard of clinical trials, 43.2% reported having little knowledge, and 75.2% expressed a desire to learn more [6]. These findings suggest that increased investment in public education and communication strategies is essential to match Poland’s clinical research infrastructure with an informed and engaged society.

Undoubtedly, the issue of recruitment to CT remains a complex process, and the success of a clinical trial largely depends on the successful recruitment of an appropriate number of participants in the subsequent phases of the study and on participant retention [7]. This process requires careful planning, direct networking, and awareness-raising among the public [8]. Research shows that patients’ preference is to be sure that the expected benefits of participation outweigh the potential risks, inconveniences, and other inconveniences related to participation in a clinical trial. For many, it is crucial to understand exactly what the benefits and risks may be in order to be able to make an informed decision as to whether to participate or not [9]. Therefore, it is crucial that researchers and recruiters actively encourage potential participants to share their concerns, provide relevant information, and support them in overcoming any arising doubts [10]. Any unresolved issues contribute to maintaining distrust, which hinders the recruitment process. Additionally, when patients refuse to participate due to unexpressed concerns, they miss out on potential benefits, such as access to high-quality diagnostics and medical care, an increased sense of security when facing illness, trust in the therapeutic team, community with other patients, an in-depth understanding of one’s health condition, and satisfaction from one’s contribution to the development of science [11,12].

In the above context, improving communication skills becomes one of the key elements in the process of enhancing research engagement [13], and according to the surveyed patients, the quality of communication significantly affects their decisions about participation in clinical trials [14]. It is worth paying attention to the relationship between the quality of communication in clinical practice and the decision-making process and considering which methods are the most appropriate for ensuring more effective communication with respect to the potential trial participants.

Many patients, when considering participation in clinical trials, are motivated by the hope of receiving health benefits or the possibility of accessing modern therapies that exceed current therapeutic procedures. Altruism and trust in healthcare professionals are cited as being among the main motivations [15,16]. Other participants, on the other hand, are driven by curiosity or interest in scientific research [17,18]. Participation in the research takes into account not only free access to medical care but also the possibility of a systematic monitoring of one’s health [19]. Some patients are also motivated by the opportunity to meet new people, socialize, and engage in an inspiring and meaningful activity [20].

Research also indicates that participants in clinical trials are motivated by a desire to help others and a belief that they fully meet the inclusion criteria of the study [12,21,22]. Some studies, however, suggest that altruism is not always the dominant motivator; when it is present, it often plays a secondary role [23]. Logistical motivations (such as time and location) are also important in the decision-making process [24].

According to the findings from a literature review, when looking at healthy people participating in CT, financial compensation is their main motivation, along with others, such as contributing to science, the health of others, and the use of additional healthcare services [25]. It is emphasized that without a financial incentive in particular, the recruitment of participants for phase I of a clinical trial would be a slow process, potentially hampering progress in the development of new medicines [26,27]. However, the results of many studies have shown that participants’ motivations are diverse and not only dominated by financial benefits, thus providing valuable information for the ethical debate on financial incentives. Having an appropriate time frame and flexible schedule and feeling comfortable participating in the study were the most frequently reported facilitators, while inflexible schedule and time commitment were the most commonly reported barriers [28]. In addition, some authors claim that people who choose not to participate in clinical trials cite the fact that they have already had the disease, concerns about changing treatments, and a lack of information about potential side effects as reasons to refuse [29].

Stakeholders involved in clinical trials see the need to increase the effectiveness of trial participant recruitment and retention, which can be fostered by improving the involvement of the public, patients, and their families [30]. Some of the relevant scientific studies, including this one, focus on identifying the opinions of not only clinical trial participants but also people who did not participate in them. Taking a long-term perspective, this allows researchers to capture the trends in changing knowledge and social attitudes. The Centre for Information and Study on Clinical Research Participation (CISCRP) conducts a global online survey every two years called the Perceptions and Insights (P&I) survey. Since 2013, this study has examined public and patient perceptions, motivations, and experiences related to clinical research. The results of such studies are extremely important because they can serve as a basis for ensuring the effective involvement of patients in clinical trials [31]. So far, beliefs and attitudes towards clinical trials, as well as general population awareness, have already been examined in Poland [6,32]. In light of the available knowledge, the research presented here is the first such large-scale research project conducted in our country. A better understanding of the reasons why individuals choose to participate in these studies will allow us to design interventions that translate into social benefits and to eliminate a number of the existing barriers to recruitment [18].

The primary aim of this study is to assess the attitudes of people benefiting from medical services, representing the general population, to participating in clinical trials. Secondary aims include identifying key sociodemographic and health-related predictors of willingness to participate, as well as analyzing the variability of the perceived health impact (CT-PHI) associated with one’s involvement in clinical trials.

## 2. Materials and Methods

### 2.1. Sample and Procedure

This survey was conducted as part of the project entitled *Humanization of the treatment process and clinical communication between patients and medical staff during the COVID-19 pandemic.* The project, carried out at the University of Warsaw, was funded by the state budget through a grant from the Medical Research Agency (2021/ABM/COVID/UW).

This quantitative cross-sectional study was conducted online between the 2nd and 20th March 2022 among individuals registered in the Redaktor.Opinii research panel. The sample was randomly drawn, in cooperation with the research team, by an external company, Interactive Research Centre sp. z o.o., which regularly uses this participant panel for survey research. The aim was to obtain a sample consistent with the structure of the Polish population covering all provinces with a predetermined target sample size. When drawing the sample, stratification by gender, age, level of education, region, and place of residence size were taken into account [33]. The inclusion criterion was the need to use medical services as a patient within the past 24 months. The exclusion criteria included employment in the healthcare sector and limiting contact with medical services, such as vaccinations, obtaining prescriptions, or other administrative procedures.

This study included a total of 2050 individuals, and patient anonymity and data protection were ensured. In this article, a significant segment of the analyses focuses on the subset of 1155 respondents who answered affirmatively to the question of whether they would be interested in participating in clinical trials in the future. The sample size, considering only those describing their approach to clinical trials, exceeds the standard sample of 1000 respondents recommended in opinion polls, and the total number of respondents (N = 2050) is twice as large. It was also verified, using G*Power 3.1.9.7 software [34], that such a sample yields a test power (1-beta) of 0.99 when comparing the mean indices of 2–4 groups, the standard alpha level (0.05), and a medium effect size (0.25).

Prior to the main study, both qualitative and quantitative pilot testing of the questionnaire were conducted to ensure the clarity, relevance, and reliability of the items.

This study received positive approval from the Research Ethics Committee of the Pedagogical Faculty of the University of Warsaw (decision number 2021/8).

### 2.2. Dependent Variables

The block of questions assessing attitudes toward clinical research was developed by the research team following a literature review and consultations with experts. It included both researcher-generated questions and items adapted from the available frameworks, such as the Study Participant Feedback Questionnaire (SPFQ), and prior studies on patient satisfaction in clinical trials. The questions were preceded by an introductory section explaining the concept of clinical research [35,36]. This section of the questionnaire identified three main thematic areas related to the decision of whether to participate in clinical trials or not. These include potential difficulties, such as, for example, those related to follow-up visits; factors related to communication and the physician–patient relationship; and health benefits or side effects. This article focuses on the third thematic area, while the findings related to the previous areas are presented and discussed in the report on the implementation of the entire project on the humanization of medical care [33].

The first key variable was interest in future participation in clinical trials. Respondents who answered affirmatively were asked about the health benefits and side effects they would consider when deciding whether to participate in, or to withdraw from, a clinical trial at the present moment. The importance of six reasons was assessed on a five-point scale ranging from “completely unimportant” to “very important”, and a summary index referring to perceived health benefits and side effects was proposed, hereinafter referred to as Clinical Trials—Perceived Health Impact (CT-PHI).

### 2.3. Independent Variables

Sociodemographic factors and factors related to health assessment and the use of medical services were taken into account when examining the determinants of interest in clinical trials and the variability of the CT-PHI index.

Sociodemographic factors:

- Gender: men and women;
- Age groups: 18–35 years old; 36–50 years old, 51–65 years old, 66 years old or older;
- Place of residence: urban and rural;
- Education level: below secondary, secondary, above secondary;
- Being in a stable relationship in categories: yes, no;
- Current employment status: yes, no;
- Financial status coded into four categories: low, average, rather high, and definitely high.

It was assessed based on a standard national question regarding one’s ability to meet basic living needs. Individuals with the lowest status could not afford to meet their most urgent needs, while those with the highest status were able to save part of their income or to invest.

#### Health-Related Factors

Self-perceived health was coded into three categories: poor (a combination of the categories rather poor and definitely poor), average (the category neither good nor bad), and good (a combination of the categories rather good and definitely good).

There were five response categories pertaining to the severity of the reduced quality of life according to the EQ-5D-5L questionnaire [37,38], including problems related to mobility; looking after oneself; doing usual activities; having pain or discomfort; and feeling worried, sad, or unhappy. The original five response categories were recoded into four, combining the two most aggravating.

The level of awareness of patient rights was assessed in three categories: no knowledge (I have never heard of patient rights), limited awareness (I have heard of patient rights, but I cannot name any), and comprehensive knowledge (I am familiar with patient rights).

Use of healthcare in the last 24 months was assessed via three response options: public only (National Health Fund), private only, and both public and private

Adherence to doctor’s recommendations was assessed via three response categories: rarely or not at all (merger of responses: sometimes, almost never, never), almost always, and always.

An additional feature used in the stratification of the analyses was the main reason given for participating in the clinical trials. Respondents were presented with the following options: financial reasons, improvement of their own health, lack of available standard treatment methods of their disease, willingness to contribute to science development and the improvement of the health of others (hereinafter referred to as altruistic considerations), and other reasons (to be entered).

The sample characteristics concerning the aforementioned factors are presented in the following tables. The characteristics of the full sample of 2050 respondents and those of the subset of 1155 individuals who expressed interest in participating in clinical trials are shown separately.

### 2.4. Data Analysis

The percentage of individuals who expressed interest in participating in clinical trials is presented according to the characteristics described above. The differences were tested with a chi-square test, interpreting the differences based on adjusted standardized residuals. A logistic model was estimated to identify independent predictors of future participation in clinical trials. The results are presented as an odds ratio (OR) with a 95% confidence interval (CI). An OR greater than 1 suggests that future participation in clinical trials is more likely in a given group when compared to the reference group, while an OR less than 1 suggests that it is less likely. When the CI includes 1, it suggests that the predictor variable has no significant effect on the decision to participate in clinical trials.

The percentage distribution of responses to the question about the main reason for one’s positive attitude towards clinical trials and the distribution of the answers provided to the six questions concerning the potential benefits and side effects of participating in clinical trials have been presented.

The psychometric properties of alternative summary CT-PHI indices were examined using confirmatory factor analysis (CFA) in order to assess the structure and reliability of both the longer and shorter scales. Reliability was evaluated using Cronbach’s alpha coefficient. Decisions regarding the elimination of specific items were based on changes in this coefficient and the values of modification indices obtained through CFA. Detailed CFA model estimation results are provided as supplementary electronic material, which also includes an explanation of the model fit parameters. The exact results of the CFA model estimation are included as supplementary electronic material, and the importance of model fitting parameters is also explained in this Appendix A. The main parameter was the root mean square error of approximation (RMSEA) indicator, for which values below 0.08 are considered acceptable, and those above 0.10 are considered poor.

The means of the CT-PHI index were compared in groups distinguished due to sociodemographic characteristics, self-assessment of health, and other factors related to the use of medical services. The differences were tested with a non-parametric Mann–Whitney (MW) or Kruskal–Wallis (KW) test depending on the number of groups compared. In the justified cases, a post hoc analysis of multiple comparisons of all group pairs for the KW test was performed.

In the last stage of the analysis, generalized linear models (GLMZ) were estimated, identifying independent predictors of CT-PHI index variability, including the previously analyzed demographic and social characteristics of respondents and factors related to health assessment and the use of medical services as independent variables. This method was adapted to the distribution of the dependent variable and the independent variables, measured on an ordinal or nominal scale. The basic model was estimated for the entire study group, and additional models were developed for subgroups stratified by participants’ main reason for interest in clinical trial participation. When presenting the results of the estimation, the values of the parameters (beta coefficient) for the explanatory variables that were included in the final model, along with their standard error (SE) and the level of significance (*p*), as well as the R-squared coefficient of determination as a goodness-of-fit statistic, were given. Beta represents the change in the expected value of the response variable for a one-unit increase in the corresponding variable, while maintaining all other independent variables as constant.

Logistic and linear models were estimated, with a full list of sixteen independent variables, and then recalculated to reduce missing data once the final solution was obtained.

Data were analyzed using SPSS^®^ version 29 (IBM, New York, NY, USA) with AMOS^®^ (29, IBM, New York). All tests were considered statistically significant at *p* ≤ 0.05.

## 3. Results

### 3.1. Sample Characteristics

The studied sample of 2050 individuals from the general population was perfectly balanced by gender, as dictated by the study’s design. The participants were aged between 18 and 86 years, with an average age of 48.8 years (SD = 16.7). The sample structure by place of residence indicated a slight over-representation of city dwellers, who accounted for 64.2% of the sample (compared to 60.7% among individuals aged 18 and over, according to nationwide data from the Central Statistical Office as of 30 June 2022) [39]. In the surveyed sample, the percentage of people with an education level above secondary was 37.4%, which included post-secondary education (9.0%). According to the data from the National Census 2021, the percentage of people with higher education (excluding post-secondary education) was 25.1% [40], which indicates that the sample is representative, given that official national data refer to the population aged 15 and over. Among the 2050 respondents, people living in a stable relationship prevailed (72.6%). The sample was almost perfectly balanced in terms of employment status; however, the share of the employed was clearly higher among men than among women (58.3% vs. 42.7%, *p* < 0.001). The structure of the wealth of the families of the surveyed respondents indicated the dominance of the average category (54.3%), and 14.9% and 13.2% of the respondents, respectively, were classified as extremely poor and definitely wealthy.

Taking into account the respondents’ health conditions, it was found that the majority assessed their health as good (47.2%), including 8.2% who assessed it as very good. The overall health-related quality of life index, assessed on the basis of the EQ-5D-5L questionnaire according to Polish standards, was 0.895 (SD = 0.134). A total of 15.7% of the respondents assessed their health status as ideal (an EQ-5D-5L index equal to 1). The severity of the health problems included in this questionnaire was assessed, and the most frequently reported issue was pain (72.0%), of which 8.8% recorded severe pain or discomfort. A significant percentage of respondents reported at least mild mental health problems, defined as anxiety or depression (66.1%), including 12.7% who declared serious problems. The third most common symptom was mobility problems, declared by 32.7% of respondents, including 4.7% who reported serious problems. A total of 23.5% of the respondents reported problems with performing daily activities, including 2.2% who declared serious problems. Problems with self-care (such as washing and dressing) were reported relatively rarely and concerned 11.7% of respondents, and in 1.2% of cases, these were serious problems. In this study, the two most burdensome categories of responses were combined, with the proportion of extremely unfavorable ratings ranging from 6 to 27 cases depending on the symptom.

Three characteristics of the respondents, which determined their functioning as patients, were also taken into account. In the last 24 months, the vast majority used medical services financed by the National Health Fund (97.6%), including 45.2% who used only public medical care and 52.4% who used both public and private care. Almost every fourth respondent (23.8%) had comprehensive knowledge about patients’ rights, but 6.4% had not heard of such rights at all. The respondents mostly claimed that they always or almost always followed the doctor’s recommendations in the treatment process (40.6% and 51.8%, respectively), which means that relatively lower adherence applied to the remaining 7.6%.

### 3.2. Interest in Participating in Clinical Trials in the Future

#### 3.2.1. Prevalence and Primary Reasons for Interest

**Figure 1:**
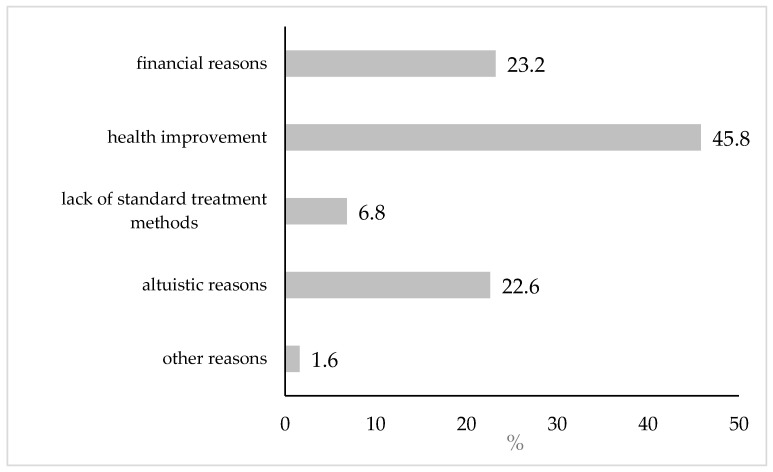
Major reasons (%) for interest in participating in clinical trials (N = 1155).

In the study group of 2050 people, more than half (56.3%) answered affirmatively to the question about their willingness to participate in clinical trials in the future. These respondents were asked to provide the main reason for such a decision, as illustrated in Figure 1. The vast majority associated their interest in clinical trials with the desire to improve their health (45.8%). The second most common choice turned out to be financial motivation (23.2%). With a similar frequency (22.7%), altruistic considerations were indicated as a way to contribute to the development of science and improve the health of others. Much less often (6.8%), the lack of standard methods of treatment of a given disease was chosen as the main motive behind the participants’ interest. In the study group, there were also 18 people who did not associate their decision with the four reasons given and marked the answer “other”. Among these answers, five can be classified as health or treatment-related reasons and one as financial. Nine people stated that they would be driven by curiosity or a desire to gain new experiences, and three considered all of the reasons given to be equivalent. The distribution of responses to the question about one’s reasons for interest in clinical trials did not differ according to the gender of the respondents (*p* = 0.379). Age turned out to be a differentiating factor (*p* < 0.001). With age, the percentage of people who chose financial reasons decreased, and the tendency to indicate health reasons increased. A relationship was also found between the reasons for interest in clinical trials and the financial situation of the family (*p* < 0.001). Individuals from the lowest socioeconomic background were less likely to indicate altruistic motivations and more likely to emphasize financial considerations.

#### 3.2.2. Univariate Analysis Determinants

Table 1 presents the percentages of people declaring interest in clinical trials according to eleven demographic, social, health, or treatment factors.

In four cases, the significance of the relationship was confirmed. People in a relationship were more likely to participate in clinical trials than those who were single. Working individuals were also more likely to participate compared to those currently unemployed. A relationship between the financial status of the family and self-assessment of health was also demonstrated, to the detriment of people from the poorest families and those who assessed their health as being worse. Table 2 provides a similar breakdown by severity of the symptoms included in the EQ-5D-5L questionnaire, which provides an in-depth analysis of the relationship between participation in clinical trials and health status. Interest in clinical trials was shown to be associated with the severity of pain and symptoms of the deterioration of one’s mental health. People with more serious health problems were much more likely to declare their willingness to participate in a clinical trial.

#### 3.2.3. Multivariate Logistic Model

Table 3 shows the results of the estimation of the logistic regression model. Four independent predictors of interest in clinical trials were identified. As the first step, the employment status was classified into the model; in the second, the severity of pain; in the third, the financial situation of the family; and the final step included being in a stable relationship. The final model did not include self-reported health and the deterioration of one’s mental health (i.e., frequent anxiety or depression), which demonstrated an association with interest in clinical trials in simple multipartite tables.

### 3.3. Perceived Health Impact

#### 3.3.1. Structure and Distribution of the CT-PHI Index

**Figure 2:**
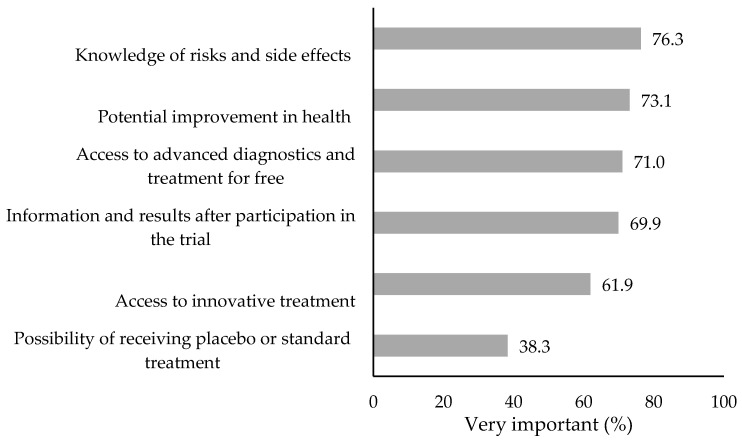
People who rated a given aspect of the clinical trial as very important (N = 1155).

The analysis of the CT-PHI component questions indicates that respondents generally considered all the reasons given to be important or very important. The percentage of the highest grade is shown in Figure 2. It is clearly the lowest in relation to the question about one’s concerns about treatment with the standard method or placebo, i.e., in the case of having no access to the more innovative therapy being tested. For other expected benefits or concerns, a given issue was assessed as very important by 61.9–76.3% of the respondents, and most often, the awareness of the risks and side effects of the treatment was considered as very important. It is worth noting that the discussed issues were very rarely considered unimportant. In the combined two least favorable categories, the answers ranged from 0.7% to 4.9%, and information on the treatment outcomes was considered the least important.

**Figure 3:**
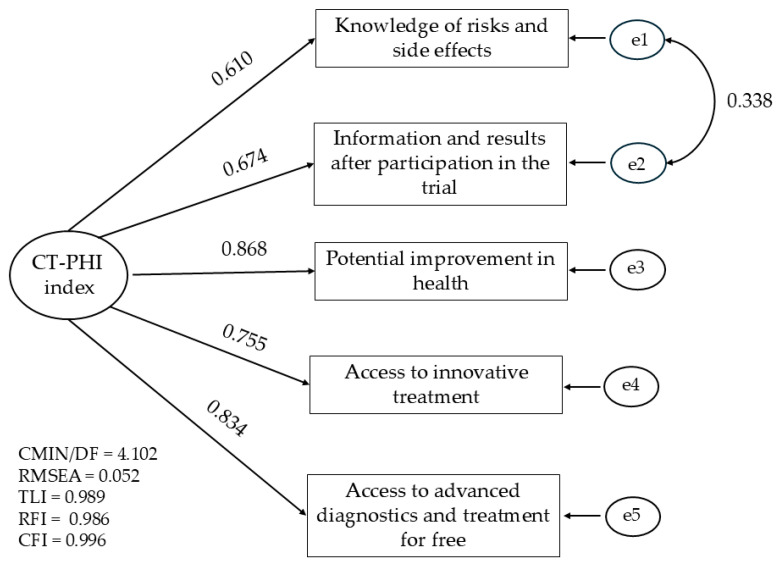
CFA model for the CT-PHI index.

An attempt was made to build a summary index on the basis of the analyzed statements. The psychometric properties of the two variants were compared. The reliability analysis of the Cronbach index indicates a satisfactory result in relation to an index having six components (alpha = 0.841). However, after excluding the placebo or standard treatment concerns statement, the reliability of the shorter index improved (alpha = 0.869). Then, the structure of the full index was compared with the index obtained after excluding the above-quoted question using the CFA method. The results are presented in (Table A1). In the first model that also considers the placebo/standard treatment concerns item, the goodness-of-fit statistics are significantly worse than in the second and third models. Both the reliability analysis and the CFA thus support the five-item model. It is characterized by extensive psychometric properties, albeit only by assuming the covariance of random components in the first two questions (Figure 3). For this model, the RMSEA fit rate = 0.052, with a 95% CI of 0.027 to 0.079. The highest factor loadings were found for the statement concerning the improvement of health and access to modern diagnostics and therapy.

The CT-PHI index based on the above model ranges from 0 to 20 points. The average is 18.16 (SD = 2.56). The distribution of CT-PHI values is left-skewed, with a skewness factor of −1.78. Almost half of the respondents rated all the analyzed aspects as very important (48.1%).

It was shown that the reason why the respondent was willing to participate in future clinical trials was the differentiation of the distribution of the CT-PHI index. The difference between the five groups distinguished by the main reason for one’s intention to participate turned out to be statistically significant in the Kruskal–Wallis test (*p* = 0.019). Multiple comparisons used for all plaintiff pairs indicate only a distinctive group of people indicating financial motivation. Respondents in this group achieved a lower average CT-PHI value than those in the group indicating health reasons (*p* = 0.003), those who pointed to the lack of alternative treatments (*p* = 0.008), and those who chose altruistic reasons (*p* = 0.032).

#### 3.3.2. CT-PHI Index Level in Univariate Analysis

Table 4 presents the distribution of CT-PHI index values in groups distinguished by eleven previously analyzed factors as potential determinants of interest in clinical trials. This allows for a comparison with the population average given previously (18.16 ± 2.56). In six cases, a statistically significant relationship was confirmed. Gender and age turned out to be strongly differentiating factors. The average CT-PHI index was higher for women than for men and steadily increased with age. In addition, the relationship with the respondent’s level of education was shown to be to the detriment of people with a less-than-secondary education. With regards to paid work, which was the main predictor of interest in clinical trials, only a trend (*p* = 0.096) was outlined, and it was in the opposite direction, i.e., in favor of the unemployed. Meanwhile, three factors related to functioning in the patient role significantly differentiated the level of the CT-PHI index. The importance of health benefits was assessed as being much less significant by people who were not aware of patient’s rights at all, use only private medical care, and have a tendency towards low adherence. The relationship between one’s self-assessment of health and one’s score was not confirmed, although in an in-depth analysis (Table 2), the level of this index changed significantly depending on the severity of the two health problems examined by the EQ-5D-5L questionnaire (limitations in self-care and pain).

#### 3.3.3. Multivariate Linear Model

Table 5 shows the results of the estimation of the generalized linear model estimated for CT-PHI, taking into account 16 potential predictors, with a reference group detailed in the footnote below the table. The final model included seven factors that explained 16% of the variability in the examined index. The factors that increased the level of CT-PHI were female gender, age over 35, living in a rural area, using both public and private healthcare services, and a high level of adherence. Regression coefficients B increased in subsequent age groups, and they were twice as high after the age of 50 than for people aged 35–50. On the other hand, the factor reducing the level of CT-PHI turned out to be average self-assessment of health and moderate problems with self-care. In both cases, no linear changes were confirmed, and only one group stood out as being to the detriment.

#### 3.3.4. Stratification by Primary Reason for Interest in Clinical Trials

We also investigated whether predictors of CT-PHI index variability fluctuated based on the previously reported reason for interest in participation in clinical trials. Three models were estimated, respectively, for those citing financial (model 1), health (model 2), and altruistic (model 3) motivations as the main reason for their interest. The other two groups were omitted due to the small number of cases. Subsequent models explain, respectively, 16.5%, 27.9%, and 5.5% of the variability of the surveyed index. A significant gender effect was maintained in models 2 and 3, and a significant age effect was maintained in all three models. However, the older group of people (36–50 years) did not differ from the reference group of the youngest adults (18–35 years). Living in rural areas only affected the variability of CT-PHI in the group of people who considered health motivation to be the main reason for their interest in participating in clinical trials. Also, only model 2 confirmed the relationship with the use of both public and private medical care. In models 1 and 2, the relationship between CT-PHI and adherence and pain was also confirmed. After stratification for the reason for one’s interest in clinical trials, a relationship with other factors, which are not shown in the general model, was revealed. Living alone was associated with a significant decrease in CT-PHI levels in model 1, and the average and rather high level of wealth contributed to an increase in this index in model 2. The severity of pain or discomfort also turned out to be an important predictor. In model 1, severe or very severe complaints were associated with an increase in CT-PHI; while in model 2, regression parameters increased with pain severity compared to the reference group of pain-free subjects. Comparing the three models presented in Table 6, it is worth noting that the largest number of factors were classified into model 2 in relation to people interested in clinical trials for health reasons. The fewest factors were identified in model 3 (altruistic reasons), where only gender and age were significant predictors, and the borderline score was obtained for the status of being in a relationship (to the detriment of singles) and with respect to adherence variable (to the advantage of always following the doctor’s recommendations).

## 4. Discussion

This study assessed patients’ attitudes toward CT participation by looking at key factors influencing their interest and the variability in perceptions of benefits and potential side effects. In this section, we will discuss sociodemographic factors, such as age, financial situation, professional activity, place of residence, marital status, and gender, and then we will analyze the factors related to health and the use of medical services that can affect this variability. However, it should be borne in mind that there is a lack of empirical research on this subject in Poland; therefore, in the discussion, we will refer primarily to the results of the research conducted in other countries. The added value of our analyses is the development of the CT-PHI index with robust psychometric properties that can be used in other studies. We qualified five of the six questions by omitting questions about the risk of treatment with a standard method or placebo. This is the aspect of clinical research that may not be understood by the broader population, and indeed, in our study, it was rated as the least significant.

### 4.1. Approach to Clinical Trials and Demographic and Social Characteristics of Respondents

In our own studies, the second most common choice in terms of interest in clinical trials turned out to be financial considerations (23.2%). Altruistic reasons were indicated with a similar frequency (22.7%). However, the vast majority of the respondents associated their interest in clinical trials with the desire to improve health (45.8%), which seems to be a general trend in Polish research, as in the Report “Awareness of Poles about clinical trials—Pratia 2022” [32]. The most important and most common motivation for participation in clinical trials among the respondents was the chance to cure diseases for which other methods failed (66%). Such motivations are also confirmed by studies in which the reasons to participate in the later phases of clinical trials, where it is indicated that the strongest factor inducing patients to participate is the prospect of personal therapeutic benefits, were analyzed [23]. Patients described the benefits of participating in CT in terms of receiving screening, learning about their own health, and improving their daily health routines [41]. Therefore, the possibility of obtaining health benefits from participation in trials should be taken into account in order to encourage patients to participate in clinical trials [42], especially in Poland, where it is the most frequently declared reason for interest in CTs.

The research performed found a relationship between one’s reasons for interest in clinical trials and one’s family financial situation. Respondents from the poorest families were less likely to indicate altruistic reasons and, more often, financial reasons. In comparison to people with a significantly high financial status, people with low status were more likely to express their intention to participate in clinical trials. People with a lower financial status may treat participation in clinical trials as an opportunity to improve their financial situation, which may explain their greater willingness to participate. U.S. studies show that individuals with lower education or those who are unemployed often view clinical trial participation primarily as a source of financial benefit, with less emphasis on its social value [41]. In addition, monetary compensation as a major motivator for participation in phase I trials was less valued by healthy volunteers with higher incomes and education [43]. It is also worth noting that healthy volunteers who were driven by financial motives took part in more clinical trials. This suggests that financial compensation may encourage them to participate repeatedly, which is worth considering when recruiting for research [15].

On the other hand, the literature indicates that people with low incomes and lower education are less willing to participate in clinical trials and are less likely to be asked to participate in clinical trials compared to people with higher incomes and higher education [44]. For example, in the case of CT, lower education and income levels are linked to lower health literacy, which may discourage physicians from informing such patients about clinical trial opportunities, often due to biased assumptions about their ability to comply with study protocols [45].

Working people were significantly more likely to express their interest in participating in clinical trials compared to those who were unemployed. This result suggests that professional activity may be associated with a greater propensity to participate in CT, which may result, among other things, from greater health awareness, financial stability, or access to information about research. Research indicates that longer periods of unemployment are associated with worsening health-promoting behaviors and health outcomes, with the worst rates reported among those unable to work [46]. These results highlight the existence of social inequalities that can affect one’s ability to participate in clinical trials, limiting access for people in more difficult financial and professional situations.

This research also shows greater openness to clinical trials among people living in rural areas, which is confirmed by other studies [47]. Taking into account the Polish situation, one of the possible explanations for this relationship is limited access to highly specialized healthcare in rural areas, which may encourage residents to look for alternative treatment options, including participation in clinical trials. However, it is worth noting that despite the greater willingness to participate, logistical barriers, such as the need to travel to study sites, may actually limit the actual participation of rural residents in clinical trials [48].

People in stable relationships also have a significantly higher tendency to declare their intention to participate in clinical trials than single people. This may suggest that the support of a life partner influences a positive attitude towards research, e.g., by encouraging people to take care of their health or by providing a greater sense of security in the context of participation in the study [49]. These results indicate the importance of information activities addressed not only at potential participants but also to their immediate environment. Incorporating the role of peer support into the recruitment strategies can increase the effectiveness of the research campaigns.

In addition, the female gender remained associated with a higher propensity to participate in clinical trials. However, it was a significant predictor only in models concerning health improvement and altruistic reasons, where women had higher levels of CT-PHI. This may be due to their greater concern for health and more frequent involvement in pro-social activities. The lack of a gender effect in model 1, on the other hand, suggests that both women and men are guided by similar considerations when it comes to financial motivation. It is worth noting here that despite regular contact with healthcare providers, women often face barriers that limit their participation in clinical trials. These barriers include increased stigma and economic and caring responsibilities that deprioritize their own health [41,50]. Therefore, it is extremely important to recognize women as a group interested in participating in clinical trials, especially since research has shown significant differences between the sexes in terms of the impact of social factors on one’s decision to participate in clinical trials. Women, more often than men, were guided by the opinions of relatives and researchers, as well as altruistic considerations [51].

The last sociodemographic variable is age. The literature emphasizes that older people have different motivations, namely, that they expect to receive the best available treatment when participating in a study [15,41], which is confirmed by the results of our own research. Our analyses show that people aged 66 or over had the highest CT-PHI index value, and with age, the percentage of people who chose financial reasons decreased and the popularity of health reasons increased. Older people, regardless of their motivation, showed the greatest openness to participating in clinical trials. Perhaps older participants are more likely to favor health benefits because they are more sensitive to the number and type of health problems that develop with age [52]; therefore, confirmation of good health through screening may be of particular importance to them.

### 4.2. Approach to Clinical Trials and the Severity of Health Problems

As far as health factors are concerned, it has been shown that interest in participating in clinical trials is associated with the severity of pain and symptoms of the deterioration of one’s mental health. People with more serious health problems were much more likely to declare their willingness to participate in a clinical trial. It is also important to recognize that chronic pain and depression can be challenging for recruitment and retention in clinical trials. In a literature review concerning clinical trial participation among patients with chronic pain, participants ranked professional relationships with study staff among the top three motivations (along with access to treatment and altruism) for study participation [21]. A declared willingness to participate does not always predict actual enrollment; only those with severe pain showed significantly higher interest, while those with moderate pain did not differ from pain-free respondents [53]. On the one hand, many patients with chronic pain have a high burden of disease and are dissatisfied with the effectiveness of available treatments, which is a factor related to their motivation to participate in clinical trials. Patients with chronic pain often experience fatigue, insomnia, depression, anxiety, and disability, and many have comorbidities that may have an impact on their willingness or ability to participate in clinical trials [54,55].

At the same time, this research has shown that people with average health ratings may be less interested in participating in studies, perhaps because they perceive their condition as stable enough not to seek additional intervention. The results of the research in this area remain inconclusive as patients who perceived themselves as being in good/excellent health were more likely to participate in clinical trials than those who reported good/moderate/poor health [56]. Other authors, however, did not note a relationship between the respondent’s perception of their health and participation in their study [57]. It may be that people with average health ratings experience difficulties balancing the potential gains and losses of participating in studies, especially if they perceive their condition as being so good that they do not presently require any interventions.

The results of our own research also suggest that moderate difficulties in self-care may negatively affect one’s willingness to participate in studies. The degree of functional independence can affect an individual’s ability to participate in clinical trials, and moderate difficulty can be a barrier to deciding whether to participate.

### 4.3. Approach to Clinical Trials and the Use of Medical Care

People using both public and private healthcare had a significantly higher CT-PHI index level than people using public care alone. This may mean that people with access to both healthcare systems are more likely to engage in clinical trials. This may be due to more contact with doctors and greater awareness of the possibility of participating in trials.

People who always followed medical recommendations had a significantly higher level of CT-PHI than those who rarely or never adhered to recommendations, though in the case of altruistic motivation, adherence plays a less significant role. These results indicate that adherence may be an important predisposing factor for participation in clinical trials, which may result from the desire to take control of one’s own health rather than being a passive recipient of medical services [58] and from the experience of better contact with doctors. This may be due to the fact that respondents who have high levels of adherence often experience better contact with their physicians. When physicians empathically acknowledge patients’ feelings and encourage them to pursue their treatment goal, patients show a reduction in anxiety symptoms and increased confidence in doctors’ recommendations [59]. The reverse effect is also possible; although studies on bias among clinicians referring patients for computed tomography scans are sparse, one qualitative study conducted among key stakeholders in several cancer clinics in the US found that clinicians were less likely to refer individuals whom they perceived as unlikely to adhere to the study protocol [60].

### 4.4. Summary of Multivariate Analysis

To sum up, the results show differences in the factors affecting CT-PHI levels depending on one’s motivation to participate in clinical trials. The largest number of relevant predictors were identified in model 2, which may mean that individuals motivated by health reasons are most susceptible to the influence of a variety of demographic and health factors. In model 1 (financial motivations), socioeconomic factors played a greater role, while in model 3, the effect on CT-PHI was the least diverse, which may suggest that altruistic individuals make the decision to participate in studies in a more homogeneous way, i.e., being less dependent on individual conditions.

### 4.5. Practical Implications for Recruitment Campaigns in Poland

Our findings suggest that recruitment campaigns in Poland should emphasize personal health benefits as this is the most common motivation for participation. Messaging should be tailored to specific demographic groups, particularly older adults, women, and rural residents, as well as to address logistical barriers to access. Additionally, increasing public awareness of patient rights and engaging close relatives in communication may enhance trust and willingness to participate in clinical trials. Involving healthcare professionals as trusted sources of information can further strengthen the engagement. Ultimately, a segmented and patient-centered approach is essential to improving recruitment effectiveness in the Polish context.

### 4.6. Strenghts and Limitations

To the best of our knowledge, the study is the first in Poland that analyzes the attitudes of the general public towards clinical trials, not being limited to people currently facing the decision to accept an invitation to participate in a CT. A broad sample of respondents, including 2050 individuals, allows for the generalization of the obtained results to the population of adult Poles using medical services. We also tested a short scale that can be implemented in future studies of this kind.

One of the main limitations of this study is its cross-sectional nature, which makes it impossible to determine cause–effect relationships. In addition, due to the self-reported nature of the data collected, there is a risk of errors resulting from the tendency of respondents to provide socially desirable answers (social desirability bias) [61], particularly concerning their motivation to participate in clinical trials. An important limitation may also be the recruitment method, which was conducted online [62], and which may have resulted in an over-representation of individuals who are more digitally active. Moreover, it was challenging to fully capture the clinical context. Participants were not asked whether they were currently or had previously participated in clinical trials, but we assume that such cases were rare. Although the introductory text explained the concept of clinical trials, it did not differentiate that these also include phase I trials involving the recruitment of healthy participants. However, the questions regarding one’s willingness to help others relate to this aspect. It was assumed that health status might influence the perception of clinical trials, but the survey did not include space for a detailed medical interview. Here, self-rated health reports and responses to the EQ-5D-5L components provide a general characterization of health status without delving into whether the respondent has any medical conditions, their severity, or their specific nature. This survey was conducted two years after the pandemic, which may have contributed to a more positive attitude toward scientific research on new therapeutic methods than before, for example, in the context of vaccine development to protect against symptoms caused by the SARS-CoV-2 virus.

A substantial amount of research on willingness to participate in clinical trials has been conducted on patients affected by a specific disease and in a defined clinical context. However, it can be assumed that patients’ attitudes are influenced by the general level of awareness in society and the prevailing norms of behavior [63]. It is worth introducing a system for monitoring normative beliefs towards clinical trials, as well as perceived barriers and facilitators, by including this topic in further cross-sectional health interview surveys. Longitudinal studies conducted in the same sample may also be of unique value; however, they require a sufficiently long follow-up period. Community-based intervention studies can also attempt to assess the effectiveness of information campaigns on CT, activities to raise awareness of patients’ rights, and programs to improve doctor–patient communication and the level of trust, all in terms of access and the principles of CT. However, this might raise some new methodological issues related to separating the control group not influenced by the intervention from the study group [64].

## 5. Conclusions

This study revealed that more than half of the respondents (56.3%) expressed their willingness to participate in clinical trials in the future, indicating a growing interest in this type of research. The primary motivation for participation was the prospect of improving health, suggesting that effective communication about potential therapeutic benefits could enhance patient engagement.

Analyses also showed that the decision to participate in clinical trials is influenced by sociodemographic factors. The introduction of the CT-PHI index allowed for a better understanding of the perceived benefits and risks, highlighting significant differences depending on participants’ primary motivation, whether health-related, financial, or altruistic. Given the diversity of motivations, the scope of information provided to the public, as well as recruitment strategies in a specific clinical situation, should consider the characteristics of different audiences or potential CT participants with respect to their social background. It is worthwhile to better explain the health benefits and risks to respondents who consider financial benefits first and foremost. These may be residents of areas with higher levels of deprivation, i.e., individuals with lower economic status and poorer health, who are often disregarded in clinical trials. Providing equal access and information about clinical trials to all population groups is regarded as a factor that improves the generalizability of trial results [65]. Our findings emphasize the necessity of strengthening educational and informational efforts regarding clinical trials, as well as optimizing recruitment processes by considering the diverse motivations of patients. This approach could contribute to increasing public trust in clinical research and improving participant engagement in clinical trials in the future [65]. Our findings emphasize the necessity of strengthening educational and informational efforts regarding clinical trials, as well as optimizing recruitment processes by considering the diverse motivations of patients. This approach could contribute to increasing public trust in clinical research and improving participant engagement in clinical trials in the future.

## Figures and Tables

**Table 1:** Interest in participation in clinical trials by selected characteristics of respondents (N = 2050).

Variables	N (%)	Interest in Participation (%)		*p* *
		Yes	No	
Gender				
*Men*	1025 (50.0)	57.8	42.2	0.197
*Women*	1025 (50.0)	54.9	45.1	
Age in years				
*18–35*	510 (24.9)	53.5	46.5	
*36–50*	552 (26.9)	60.3	39.7	0.096
*52–65*	588 (28.7)	56.8	43.2	
*66+*	400 (19.5)	53.8	46.3	
Residence				
*Urban*	1316 (64.2)	56.5	43.5	0.886
*Rural*	734 (35.8)	56.1	43.9	
Education				
*Below secondary*	556 (27.1)	55.8	44.2	
*Secondary*	728 (35.5)	56.2	43.8	0.910
*Above secondary*	766 (37.4)	56.9	43.1	
Being in a stable relationship				
*Yes*	1466 (72.6)	58.0	42.0	**0.023**
*No*	553 (27.4)	52.4	47.6	
Occupational work				
*Yes*	1027 (50.5)	59.4	40.6	**0.005**
*No*	1005 (49.5)	53.2	46.8	
Financial status				
*Low*	298 (14.9)	62.8	37.2	
*Average*	1087 (54.3)	57.8	42.2	**0.016**
*Rather high*	353 (17.6)	51.6	48.4	
*Definitely high*	265 (13.2)	52.8	47.2	
Self-perceived health				
*Poor*	442 (21.6)	62.9	37.1	
*Average*	638 (31.2)	53.9	46.1	**0.008**
*Good*	964 (47.2)	55.2	44.8	
Knowing the rights of patients				
*No knowledge*	135 (6.6)	54.8	45.2	
*Limited awareness*	1445 (70.5)	55.8	44.2	0.545
*Comprehensive knowledge*	470 (22.9)	58.5	41.5	
Healthcare				
*Public only*	900 (43.9)	58.0	42.0	
*Private only*	61 (3.0)	45.9	54.1	0.137
*Both public and private*	1089 (53.1)	55.6	44.4	
Adherence to treatment				
*Rarely or not at all*	148 (7.2)	59.5	40.5	
*Almost always*	1064 (52.1)	56.1	43.9	0.740
*Always*	831 (40.7)	56.3	43.7	

* chi-square test; statistical significance in bold.

**Table 2:** Interest in participation in clinical trials and CT-PHI index level depending on the severity of problems according to EQ-5D-5L.

Problems with:	N (%) *	Interest in Participation (%) (N = 2050)			CT-PHI Index (N = 1155)	
		Yes	No	*p* **	M ± SD	*p* ***
Mobility						
*No problems*	1380 (67.3)	54.7	45.3		18.22 ± 2.47	
*Slight problems*	379 (18.5)	59.4	40.6	0.124	18.07 ± 2.74	0.885
*Moderate problems*	195 (9.5)	57.9	42.1		17.99 ± 2.87	
*Severe problems/incapacity*	96 (4.7)	64.6	35.4		18.16 ± 2.46	
Self-care						
*No problems*	1810 (88.3)	55.5	44.5		18.26 ± 2.48	
*Slight problems*	144 (7.0)	61.1	38.9	0.190	17.99 ± 2.74	**<0.001**
*Moderate problems*	71 (3.5)	66.2	33.8		16.53 ± 3.30	
*Severe problems/incapacity*	25 (1.2)	60.0	40.0		17.80 ± 2.88	
Usual activities						
*No problems*	1569 (76.5)	54.9	45.1		18.23 ± 2.49	
*Slight problems*	294 (14.4)	61.2	38.8	0.136	18.27 ± 2.41	0.272
*Moderate problems*	141 (6.9)	59.6	40.4		17.40 ± 3.29	
*Severe problems/incapacity*	46 (2.2)	63.0	37.0		17.76 ± 2.92	
Pain/discomfort						
*No*	573 (28.0)	54.5	45.5		17.76 ± 2.87	
*Slight*	774 (37.7)	53.6	46.4	**0.011**	18.23 ± 2.43	**0.014**
*Moderate*	523 (25.5)	59.3	40.7		18.40 ± 2.30	
*Severe/extreme*	180 (8.8)	65.6	34.3		18.37 ± 2.69	
Anxiety/depression						
*No*	694 (33.9)	53.7	46.3		18.17 ± 2.68	
*Slight*	710 (34.6)	55.9	44.1	**0.043**	18.22 ± 2.40	0.445
*Moderate*	385 (18.8)	56.6	43.4		18.26 ± 2.45	
*Severe/extreme*	261 (12.7)	64.0	36.0		17.88 ± 2.80	

* The structure given for a total sample; ** chi-square test; *** non-parametric Kruskal–Wallis test; statistical significance in bold.

**Table 3:** Estimation of logistic regression model * for interest in participation in clinical trials (N = 2050).

Independent Variables	B	SE(B)	*p*	OR	95% CI(OR)
Occupational work					
Yes	**0.296**	**0.094**	**0.002**	**1.345**	**1.119–1.616**
No (ref.)				1.000	
Being in stable relationship					
Yes	**0.215**	**0.104**	**0.038**	**1.240**	**1.012–1.520**
No (ref.)				1.000	
Financial status			0.028		
Low	**0.353**	**0.178**	**0.047**	**1.423**	**1.004–2.017**
Average	0.167	0.141	0.234	1.182	0.898–1.557
Rather high	−0.105	0.165	0.523	0.900	0.651–1.244
Definitely high (ref.)				1.000	
Pain/discomfort (EQ-5D-5L)			0.015		
No (ref.)				1.000	
Slight	−0.021	0.115	0.858	0.980	0.783–1.226
Moderate	0.175	0.128	0.169	1.192	0.928–1.530
Severe/extreme	**0.517**	**0.189**	**0.006**	**1.677**	**1.159–2.426**

* statistical significance in bold.

**Table 4:** Mean CT-PHI index by selected characteristics of respondents who declared willingness to participate in clinical trials (N = 1155).

Variables	N (%)	M ± SD	*p* *
Gender			
*Men*	592 (51.3)	17.85 ± 2.72	**<0.001**
*Women*	563 (48.7)	18.49 ± 2.35	
Age in years			
*18*–*35*	273 (23.6)	17.24 ± 2.91	
*36*–*50*	333 (28.9)	17.92 ± 2.73	**<0.001**
*52*–*65*	334 (28.9)	18.70 ± 2.27	
*66+*	215 (18.6)	18.87 ± 1.69	
Residence			
*Urban*	743 (64.3)	18.05 ± 2.71	0.115
*Rural*	412 (35.7)	18.37 ± 2.27	
Education			
*Below secondary*	310 (26.8)	17.75 ± 2.94	
*Secondary*	409 (35.5)	18.33 ± 2.45	**0.004**
*Above secondary*	436 (37.7)	18.30 ± 2.35	
Being in a stable relationship			
*Yes*	851 (74.6)	18.18 ± 2.61	0.391
*No*	290 (25.4)	18.09 ± 2.44	
Occupational work			
*Yes*	610 (53.3)	18.00 ± 2.74	0.095
*No*	535 (46.7)	18.37 ± 2.30	
Financial status			
*Low*	187 (16.4)	18.28 ± 2.50	
*Average*	628 (55.3)	18.27 ± 2.49	0.252
*Rather high*	182 (16.0)	17.84 ± 2.80	
*Definitely high*	140 (12.3)	18.08 ± 2.44	
Self-perceived health			
*Poor*	278 (24.1)	18.35 ± 2.40	
*Average*	344 (29.8)	17.96 ± 2.81	0.368
*Good*	532 (46.1)	18.21 ± 2.44	
Knowing the rights of patients			
*No knowledge*	74 (6.4)	17.22 ± 3.32	
*Limited awareness*	806 (69.8)	18.09 ± 2.63	**<0.001**
*Comprehensive knowledge*	275 (23.8)	18.64 ± 2.00	
Healthcare			
*Public only*	522 (45.2)	17.98 ± 2.68	
*Private only*	28 (2.4)	16.36 ± 4.00	**<0.001**
*Both public and private*	605 (52.4)	18.40 ± 2.32	
Adherence to treatment			
*Rarely or not at all*	88 (7.6)	16.01 ± 3.67	
*Almost always*	597 (51.8)	18.08 ± 2.39	**<0.001**
*Always*	468 (40.6)	18.71 ± 2.22	

* non-parametric test: Mann–Whitney U or Kruskal–Wallis; * statistical significance in bold.

**Table 5:** Multivariate generalized linear model for CT-PHI index (N = 1152).

Independent Variable *	B	SE(B)	*p* **
Constant	15.263	0.334	**<0.001**
*Female*	0.517	0.139	**<0.001**
Age in years			
*66 or more*	1.554	0.221	**<0.001**
*51*–*65*	1.418	0.197	**<0.001**
*36*–*50*	0.630	0.192	**0.001**
Residence—rural	0.326	0.147	**0.026**
Self-perceived health			
*Good*	−0.128	0.182	0.484
*Average*	−0.478	0.193	**0.013**
Healthcare			
*Both public and private*	0.451	0.139	**0.001**
*Only private*	−0.191	0.474	0.687
Adherence to treatment			
*Always*	2.071	0.277	**<0.001**
*Almost always*	1.734	0.269	**<0.001**
Self-care problems (EQ-5D-5L)			
*Severe/incapacity*	−0.443	0.611	0.468
*Moderate*	−1.485	0.356	**<0.001**
*Slight*	−0.443	0.267	0.097

* Reference categories: male gender, age 18–35, urban residence, poor self-perceived health, public healthcare only, rare or no adherence to treatment at all, and no self-care problems; ** statistical significance in bold.

**Table 6:** Multivariate generalized linear model for CT-PHI index in subgroups of respondents.

Independent Variables *	Financial Reasons (N = 260)			Health Improvement (N = 511)			Altruistic Reasons (N = 254)		
	B	SE(B)	*p* **	B	SE(B)	*p* **	B	SE(B)	*p* **
Constant	16.75	0.99	**0.000**	14.46	0.59	**0.000**	16.09	1.39	**0.000**
Gender female	−0.01	0.35	0.971	0.41	0.20	**0.034**	0.88	0.32	**0.006**
Age in years									
*66 or more*	2.02	0.77	**0.009**	1.24	0.34	**0.000**	1.17	0.57	**0.040**
*51*–*65*	1.21	0.48	**0.011**	1.09	0.30	**0.000**	0.99	0.46	**0.032**
*36*–*50*	0.38	0.38	0.314	0.31	0.28	0.265	0.34	0.44	0.440
Residence—rural	0.46	0.34	0.176	0.41	0.19	**0.033**	0.18	0.33	0.595
Being single	−1.11	0.41	**0.007**	0.15	0.21	0.486	−0.64	0.36	0.073
Financial status									
*Definitely high*	0.15	0.64	0.813	−0.10	0.36	0.780	−0.65	0.67	0.329
*Rather high*	−0.65	0.57	0.259	−0.73	0.34	**0.031**	−0.63	0.62	0.311
*Average*	−0.32	0.41	0.442	−0.56	0.26	**0.033**	0.04	0.55	0.940
Heath care									
*Both public and private*	0.08	0.34	0.814	0.64	0.18	**0.001**	−0.18	0.32	0.587
*Only private*	0.82	0.85	0.340	−0.33	0.77	0.664	−0.66	0.99	0.504
Adherence to treatment									
*Always*	1.66	0.57	**0.003**	2.50	0.39	**0.000**	1.36	0.72	0.060
*Almost always*	1.66	0.54	**0.002**	2.21	0.38	**0.000**	0.75	0.73	0.302
Self-care (EQ-5D-5L)									
*Severe problems/incapacity*	0.57	1.59	0.720	−3.12	1.09	**0.004**	−4.55	3.57	0.202
*Moderate problems*	−1.33	1.06	0.210	−2.54	0.59	**0.000**	0.70	1.43	0.627
*Slight problems*	−1.96	0.93	**0.035**	−0.49	0.36	0.176	0.93	0.92	0.313
Pain/discomfort (EQ-5D-5L)									
*Severe/extreme*	2.57	0.97	**0.008**	0.91	0.40	**0.023**	−0.72	0.82	0.380
*Moderate*	0.25	0.51	0.616	1.36	0.31	**0.000**	0.04	0.48	0.929
*Slight*	0.09	0.41	0.827	1.02	0.27	**0.000**	−0.22	0.38	0.565

* Reference categories: male gender, age 18–35, urban residence, being in stable relationship, low financial status, only public healthcare only, rarely or not adherent to treatment at all, no self-care problems; ** statistical significance in bold.

## Data Availability

The data are owned by Warsaw University and are not to be made freely publicly available.

## Appendix A

**Table A1:** CFA models for alternative CT-PHI indices.

	Model 1	Model 2	Model 3
Number of items	6	5	5
Covariance between residuals	-	-	e1 & e3
CMIN/DF	19.44	26.50	4.10
RMSEA—Root Mean Square Error of Approximation	0.126	0.149	0.052
95% CI (RMSEA)	0.110–0.143	0.127–0.171	0.027–0.079
NFI—Normed Fit Index	0.944	0.954	0.994
RFI—Relative Fit Index	0.906	0.907	0.986
TLI—Tucker Lewis index	0.910	0.910	0.989
CFI—Comparative Fit Index	0.946	0.955	0.996
Modification indices			
Number	12	5	3
Range	4.19–97.55	6.20–108.17	4.42–6.15
Standardized regression weights			
Item1 <- CT-PHI	0.658	0.649	0.610
Item2 <- CT-PHI	0.427	-	-
Item3 <- CT-PHI	0.713	0.707	0.674
Item4 <- CT-PHI	0.858	0.862	0.868
Item5 <- CT-PHI	0.750	0.747	0.755
Item6 <- CT-PHI	0.818	0.824	0.834

Items:

- Awareness of risks and side effects;
- Possibility of receiving placebo or standard treatment;
- Information and results after participation in the trial;
- Potential improvement in health;
- Access to innovative treatment;
- Access to advanced diagnostics and treatment for free.
Responses: very important; rather important; neither important nor unimportant; rather unimportant; not important at all (reverse coding for CT-PHI index development).
